# Association Between Metformin Use and the Risk, Prognosis of Gynecologic Cancer

**DOI:** 10.3389/fonc.2022.942380

**Published:** 2022-07-11

**Authors:** Kui Yao, Heng Zheng, Tao Li

**Affiliations:** Department of Obstetrics and Gynecology, Key Laboratory of Birth Defects and Related Diseases of Women and Children (Sichuan University), Ministry of Education, West China Second University Hospital, Sichuan University, Chengdu, China

**Keywords:** gynecologic cancer, meta-analysis, metformin, risk, prognosis

## Abstract

**Background:**

For gynecological cancer patients, the beneficial effect of metformin use remains controversial due to inconsistent results of published articles. By conducting a meta-analysis, we aimed to evaluate the effect of metformin in reducing the risk and improving the survival of gynecological cancer among women with diabetes mellitus (DM).

**Methods:**

Articles exploring association between metformin use and the risk, as well as prognosis of gynecologic cancer in DM, were searched in the databases: PubMed, Web of Science, SCOPUS, EMBASE, EBSCO, and PROQUEST. Articles were published before May 2022. All the studies were conducted using STATA 12.0 software.

**Results:**

The meta-analysis showed no significant association between metformin use and risk of gynecologic cancer in DM with a random effects model [odds ratio (ORs)/relative risk (RR) = 0.91, 95% confidence intervals (CI) 0.77 to 1.08, I^2^ = 84.2%, *p* < 0.001]. Metformin use was associated with reduced overall survival (OS) and progression-free survival (PFS) of gynecologic cancer in DM with random effects models [OS: hazard ratio (HR) = 0.60, 95% CI 0.49–0.74, I^2^ = 55.2%, *p* = 0.002; PFS: HR = 0.55, 95% CI 0.33–0.91, I^2^ = 69.1%, *p* = 0.006], whereas no significant association was showed between metformin use and recurrence-free survival (RFS), as well as cancer-specific survival (CSS) of gynecologic cancer in DM with random effects models (RFS: HR = 0.60, 95% CI 0.30–1.18, I^2^ = 73.7%, *p* = 0.010; CSS: HR = 0.78, 95% CI 0.43–1.41, I^2^ = 72.4%, *p* = 0.013).

**Conclusions:**

In conclusion, this meta-analysis indicated that metformin may be a useful adjuvant agent for gynecological cancer with DM, especially for patients with ovarian cancer and endometrial cancer.

## Introduction

Cancer has become a more and more serious problem in public sanitation globally that contributes to heavy disease burden as the second highest cause next to cardiovascular diseases (1). Cervical cancer is the most common gynecological cancer that affects more than half a million women and causes over 300,000 deaths every year (2). Due to the implementation of vaccination and cytological screening, the incidence and mortality have been declining; however, there have been differences between high-income and low-income countries (3, 4). In low-income countries, cervical cancer is still the leading cause of death related to cancer among women (5). Compared to women in UK and USA, women in China are more likely to have cervical cancer (6). The incidence of endometrial cancer is increasing, and endometrial cancer is often diagnosed in more and more young women (7, 8). Ovarian cancer is the second most common cause of death among gynecologic cancer patients with almost 140,000 deaths per year (9, 10). Thus, due to the large population, gynecologic cancers tend to be a severe public health problem in the developing countries including China. Effective prevention and therapy for gynecologic cancer are essential for public health development.

Metformin is one of the first-line drug for type 2 diabetes mellitus (T2DM), which has been used for over 60 years due to its safety and low cost (11). The stimulation of Adenosine 5‘-monophosphate (AMP)-activated protein kinase (AMPK) is the major mechanism of metformin, then metformin can inactive the mammalian target of rapamycin (mTOR) signaling via AMPK-dependent action, and mTOR has been considered as a central signaling pathway that controls cell growth and metabolism in cancer (12, 13). Moreover, mTOR complex 1 (mTORC1) and reactive oxygen species (ROS) are AMPK-independent mechanisms of metformin (14).

Considering that diabetes is a risk factor of cancer, the potential association between metformin use and cancer prevention and treatment leads to an increasing interest. In the past years, epidemiological studies and clinical trials supported that some cancers, such as head and neck (15), breast (16), pancreatic (17), colorectal (18), and liver (19), have raised the interest on the anticarcinogenic effects of metformin. Furthermore, experimental studies have been made to understand the mechanisms that underlie the anticarcinogenic effects of metformin, as an adjunct drug in the long-term management of gynecologic cancer. Lengyel et al. (20) concluded that metformin changes metabolism in ovarian cancer cells and prevents tumor growth in vitro and in mouse models. Rattan et al. (21) reported that, in addition to inhibiting tumor cell proliferation, metformin use inhibits both angiogenesis and metastatic spread of ovarian cancer in vivo. These studies provide a strong rationale for metformin use in gynecological cancer treatment. In addition, these preclinical studies suggest that metformin warrants further exploration for use as a gynecological cancer therapy. Recent epidemiological studies showed that the use of metformin can significantly decrease the risk and improve the outcome of certain cancers including gastric cancer and pancreatic cancer (22, 23). However, for gynecological cancer patients, the beneficial effect of metformin use remains controversial due to inconsistent results of published articles. Regarding association between metformin use and risk of gynecological cancer, Tseng et al. (24) found that metformin use is associated with a decreased risk of ovarian cancer. However, Bodmer et al. (25) reported that long-term use of metformin was not associated with a risk of ovarian cancer. Becker et al. (26) reported that metformin use and other antidiabetic drugs were not associated with an altered risk of endometrial cancer. Regarding association between metformin use and prognosis of gynecological cancer, Deng et al. (27) found that both overall survival (OS) and progression-free survival (PFS) of T2DM patients who took metformin were significantly prolonged compared with those of T2DM patients who did not take metformin in endometrial cancer. Hanprasertpong et al. (28) demonstrated that metformin use was associated with improved disease-free survival (DFS) in patients with cervical cancer with T2DM. However, Seebacher et al. (29) found that metformin was not associated with prolonged recurrence-free survival (RFS) or cancer-specific survival (CSS) of endometrial cancer. Garcia et al. (30) reported that no statistically significant association was observed between metformin use and OS of 360 ovarian cancer patients. Takiuchi et al. (31) reported that metformin use was not associated with survival of women with cervical cancer. Meta-analyses comparing the incidence of gynecologic cancer in diabetics using metformin with those using insulin or other anti-diabetic agents have shown somewhat variable results (32–34). In addition, up to now, no meta-analysis was made to explore the association between metformin use and the prognosis of gynecologic cancer. By conducting a meta-analysis, we aimed to evaluate the effect of metformin in reducing the risk and improving the survival of gynecological cancer among women with DM.

## Methods

The present study was made according to the Preferred Reporting Items for Systematic reviews and Meta-Analysis (PRISMA) guideline (35).

### Search Strategy

Articles exploring association between metformin use and the risk, as well as prognosis of gynecologic cancer in DM, were searched in the databases: PubMed, Web of Science, SCOPUS, EMBASE, EBSCO, and PROQUEST. Articles were published before 11 May 2022. These search terms were used: (“metformin”) AND (“gynecologic cancer” OR “ovarian cancer” OR “oophoroma“ OR “ovary carcinoma” OR “carcinoma of the ovary” OR “endometrial cancer” OR “endometrial carcinoma” OR “carcinoma of the endometrium” OR “endometrial carcinoma of the uterus” OR “cervical cancer” OR “cervical carcinoma” OR “carcinoma of the uterine cervix”). Search query was shown in **Supplementary Table 1**.

### Inclusion and Exclusion Criteria

N = 1,292 records were screened after removing N = 4,085 duplicates. Studies were included on the basis of these criteria (1): included studies should explore the association between metformin use and the risk of gynecologic cancer in DM and (2) included studies should explore the association between metformin use and prognosis of gynecologic cancer in DM. Exclusion criteria included the following (1): reviews, meta-analyses, and case reports were excluded and (2) only articles written in English were included. After exclusion, N = 169 full-text articles were accessed for eligibility. In addition, studies were excluded according to the following exclusion criteria (1): included studies should provide sufficient information for odds ratios (ORs) in case-control studies or relative risks (RRs) in cohort studies and their 95% confidence intervals (CIs) regarding association between metformin use and risk of gynecologic cancer in DM and (2) included studies should provide sufficient information for hazard ratios (HRs) and 95% CIs regarding association between metformin use and clinical outcome of gynecologic cancer in DM. Finally, N = 31 articles were included.

### Data Extraction

The following data were extracted: author, publication year, study design, study location, sample sizes of participants, mean age of participants, sample sizes of cancer cases, cancer type, adjusted variables, and results.

### Statistical Analysis

ORs/RRs or HRs and their CIs were computed. Q test and I2 were used to explore heterogeneities between included studies. When heterogeneity was low (p-value for Q test > 0.05 and I2 < 50%), fixed effects models were used; when heterogeneity was high (p-value for Q test ≤ 0.05 and I2 ≥ 50%), random effects models were used. Meta-regression analysis was conducted to explore source of heterogeneity. Subgroup studies (for different cancer types) were made to explore the source of the heterogeneity. In specific types of cancer, subgroup studies (for different ethnicities and study types) were made to explore the source of the heterogeneity. Sensitivity analysis was used to explore the study stabilization. The Begg’s test, Egger’s test, and funnel plot were used to assess publication bias. All the studies were conducted using STATA 12.0 software.

### Risk of Bias

Quality appraisal was made using the Cochrane Risk of Bias Tool. Data were analyzed using Review Manager 5.3.

## Results

### Study Characteristics

**Figure 1:**
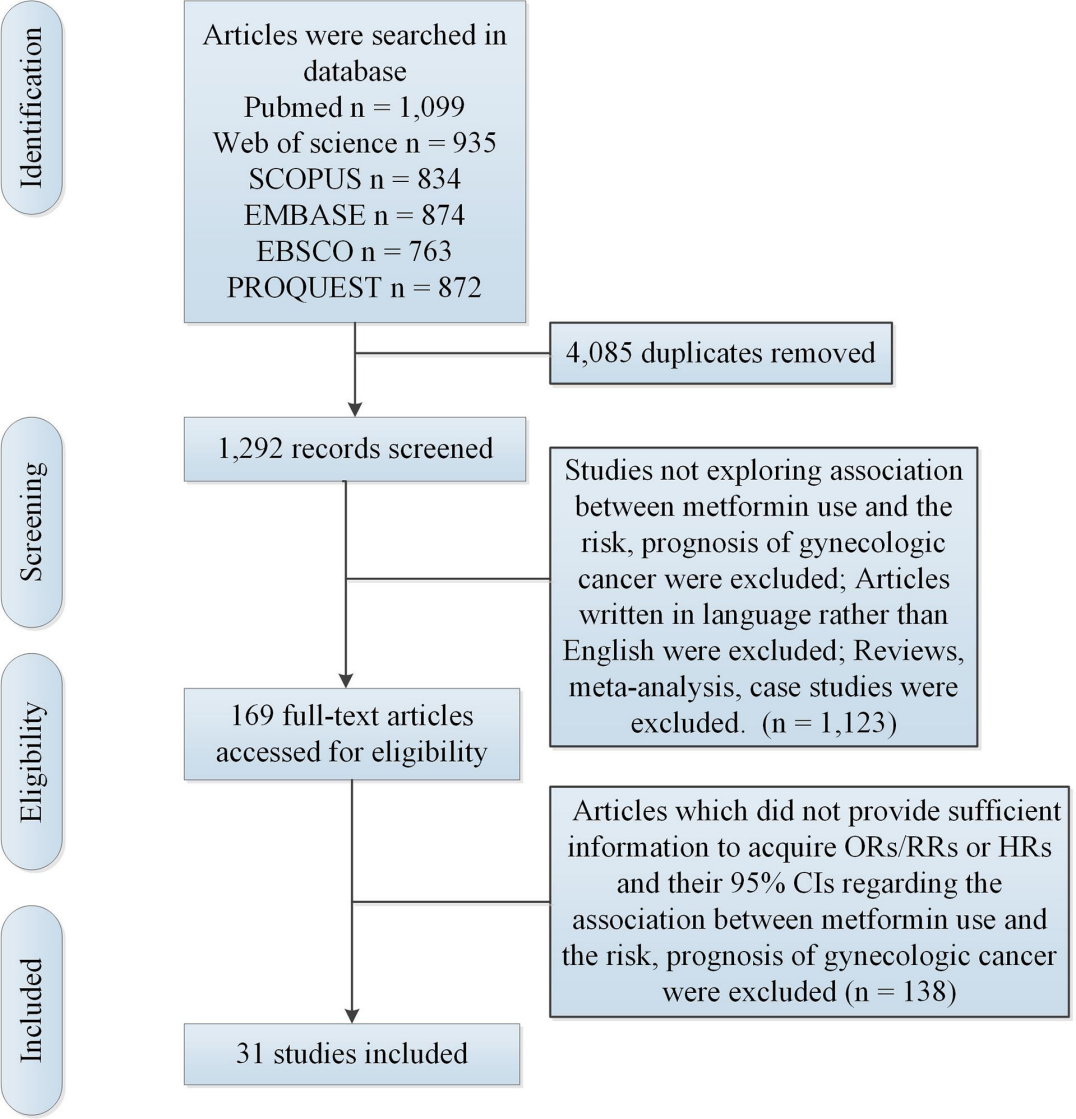
Search and selection process.

**Figure 1** illustrated the gradual selection procedures. **Tables 1**, **2** showed study characteristics. N = 11 studies (24–26, 36–39, 41, 45, 47, 48) (including 2,059,913 participants) explored the association between metformin use and risk of gynecologic cancer in DM. N = 20 studies (27–31, 40, 42–44, 46, 49–58) (including 122,738 participants) explored the association between metformin use and prognosis of gynecologic cancer in DM.

**Table 1:** Characteristics of all included studies regarding association between metformin use and risk of gynecologic cancer.

References	Study design	Country	Sample size/ mean age	Cancer cases	Cancer type	a history of metformin use before the cancer diagnosis	continued the metformin use during treating cancer	BMI	waist	blood glucose	hyperlipidemia	Adjusted variables	Results (OR/RR, 95%CI)
Becker et al. 2013 (18)	Case-control	UK	17878/ 63.0	2554	EC	Yes	NR	NR	NR	DM	NR	BMI, smoking, DM	OR: 0.86 (0.63-1.18)
Luo et al. 2014 (24)	Cohort	USA	88107/ 63.0	1241	EC	Yes	Yes	NR	NR	DM	NR	Age, BMI, race, education, smoking, physical activity, alcohol intake, HRT, oral contraception use, parity, age at first birth, different treatment assignments for clinical trials	RR: 1.64 (0.92-2.91)
KO et al. 2015 (26)	Cohort	USA	541128/ NR	729	EC	Yes	Yes	NR	NR	DM	NR	Age, Charlson index, fibroid, infertility, PCOS, DM, hypertension, endometrial hyperplasia, connective tissue disease, oral contraception use, HRT, ultrasound	RR: 1.09 (0.88-1.35)
Tseng et al. 2015 (27)	Cohort	Taiwan, China	478921/55.6	2885	EC	Yes	Yes	NR	NR	DM	NR	Age, hypertension, COPD, stroke, heart disease, obesity, metabolic profiles, various drugs	RR: 0.68 (0.61-0.74)
Franchi et al.2016 (28)	Case-control	Italy	7861/ 64	376	EC	Yes	NR	NR	NR	DM	NR	Age, date at cohort entry, duration of follow-up, the Charlson comorbidity index, cardio/cerebrovascular diseases, various drugs, HRT, oral contraception use	OR: 0.99 (0.80-1.23)
Gong et al. 2016 (19)	Cohort	USA	145826/ NR	993	EC	Yes	Yes	NR	NR	DM	NR	Age, race, education, smoking, physical activity, aspirin, hyperlipidemia, HRT, BMI, WHR	RR: 1.24 (0.90-1.70)
Arima et al. 2017 (20)	Case-control	Finland	12382/ NR	590	EC	Yes	NR	NR	NR	DM	NR	Age, DM, various drugs	OR: 1.24 (1.02-1.51)
Bodmer et al. 2011 (21)	Case-control	UK	10781/ 61.2	1611	OC	Yes	NR	NR	NR	DM	NR	BMI, smoking, HRT, oral contraception use, history of hysterectomy, endometriosis and PCOS	OR: 0.61 (0.30-1.25)
Tseng et al. 2015 (22)	Cohort	Taiwan, China	479475/ 55.6	3201	OC	Yes	Yes	NR	NR	DM	NR	Age, hypertension, COPD, stroke, obesity, eye disease, nephropathy, ischemic heart disease, peripheral arterial disease, dyslipidemia, urinary tract disease, other cancers, various drugs	RR: 0.66 (0.59-0.73)
Gong et al. 2016 (19)	Cohort	USA	145826/ NR	553	OC	Yes	Yes	NR	NR	DM	NR	Age, race, education, smoking, physical activity, aspirin use, hyperlipidemia, HRT, BMI, WHR	RR: 1.06 (0.64-1.74)
Urpilainen et al. 2018 (23)	Cohort and case-control	Finland	137643/ NR	303	OC	Yes	Yes	NR	NR	DM	NR	Age, duration of DM	RR: Cohort: 1.02 (0.72-1.45) Case-control: 0.91 (0.61-1.34)
Tseng et al. 2016 (25)	Cohort	Taiwan, China	139911/ 58.2	476	CC	Yes	Yes	NR	NR	DM	NR	Age, hypertension, COPD, stroke, obesity, eye disease, nephropathy, ischemic heart disease, peripheral arterial disease, dyslipidemia, urinary tract disease, other cancers, various drugs	RR: 0.56 (0.40-0.78)

OR, odds ratio; RR, relative risk; CI, confidence interval; NR, not reported; OC, ovarian cancer; EC, endometrial cancer; CC, cervical cancer; BMI, body mass index; DM, diabetes mellitus; HRT, hormone replacement therapy; PCOS, polycystic ovarian syndrome; COPD, chronic obstructive pulmonary disease; WHR, waist-to-hip ratio.

**Table 2:** Characteristics of all included studies regarding association between metformin use and prognosis of gynecologic cancer.

References	Study design	Country	Sample size/ mean age	Cancer type	a history of metformin use before the cancer diagnosis	continued the metformin use during treating cancer	Follow-up time, median (months)	BMI of metformin users	waist of metformin users	blood glucose of metformin users	hyperlipidemia of metformin users	Adjusted variables	Results (HR, 95%CI)
KO et al. 2014 (36)	Cohort	USA	363/ 63.4	EC	Yes	Yes	33	38 (33–46)	NR	DM	NR	Age, stage, grade, histology, adjuvant treatment	HR: RFS: 0.56 (0.34-0.91) OS: 0.43 (0.24-0.77)
Nevadunsky et al. 2014 (37)	Cohort	USA	985/ 63.9	EC	Yes	Yes	40	34.8 (6.7)	NR	DM	63 (55.3)	Age, stage, grade, radiation, chemotherapy, hyperlipidemia	OS: HR: Endometrioid: 0.79 (0.31-2.00) non-endometrioid: 0.54 (0.30-0.97)
Lemanska et al. 2015 (29)	Cohort	Poland	107/ 64.3	EC	Yes	Yes	NR	NR	NR	DM	NR	Age, BMI, grade, stage, DM, EC type, hypertension, glucose level, hysterectomy, radiation	OS: HR: 1.08 (0.46-2.56)
Al Hilli et al. 2016 (30)	Cohort	USA	1303/ 64.6	EC	Yes	Yes	51.6	39.0 (9.5)	NR	DM	NR	Age, BMI, smoking, cardiopulmonary state, ASA score, various tumor features, surgery, adjuvant therapy	HR: OS: 0.61 (0.30-1.23) PFS: 1.06 (0.34-3.30)
Hall et al. 2016 (35)	Cohort	USA	351/ 58	EC	Yes	Yes	NR	44.0	NR	DM	NR		RFS: OR: 0.17 (0.02-0.94)
Ezewuiro et al. 2016 (34)	Cohort	USA	349/ 63.3	EC	Yes	Yes	37	35.3±9.7	NR	DM	NR	study site, stage, age at chemotherapy	OS: HR: 0.42 (0.23-0.78)
Seebacher et al. 2016 (38)	Cohort	Austria	465/65.3	EC	Yes	Yes	51	35.3 (10.1)	NR	DM	NR	Age, tumor stage, grade, histological subtype	HR: RFS: 1.2 (0.8-1.7) CSS: 1.18 (0.7-1.9) OS: 0.9 (0.69-1.2)
Insin et al. 2018 (39)	Cohort	Thailand	212/ 60.2	EC	Yes	Yes	47	NR	NR	DM	NR	NR	HR: PFS: 0.47 (0.18-1.16) OS: 1.01 (0.58-1.79)
Deng et al. 2020 (31)	Cohort	China	136/ 57.0	EC	Yes	Yes	48.6	32.82±4.48	NR	DM	NR	Age, BMI, DM, FIGO stage, histologic grade, muscular invasion, lymph node metastasis	OR: OS: 0.46 (0.30-0.93) PFS: 0.41 (0.21-0.87)
Romero et al. 2012 (40)	Cohort	USA	341/ 59.7	OC	Yes	Yes	63	33.83±5.64	NR	DM	NR	Age, BMI, creatinine, FIGO stage, tumor grade, residual implants >1 cm after surgery, and histological subtype, ASA class, ethnicity, history of cardiovascular disease	HR: PFS: 0.38 (0.16-0.90) OS: 0.43 (0.16-1.19)
Currie et al. 2012 (41)	Cohort	UK	112408/ 67.8	OC and EC	Yes	Yes	19.2-24	30.7 ± 5.1	NR	DM	NR	Age, smoking, Townsend index of deprivation, Charlson comorbidity index, number of primary care contacts, year of diagnosis	OS: HR: 0.48 (0.28-0.81)
Kumar et al. 2013 (42)	Case-control	USA	215/ 60.4	OC	Yes	Yes	NR	33 ± 7	NR	DM	NR	Age, diagnosis year, BMI, stage, histology, chemotherapy, grade	OS: HR: 0.37 (0.19-0.71)
Bar et al. 2016 (43)	Cohort	Israel	143/ 62.5	OC	Yes	Yes	48.8	NR	NR	DM	NR	Age, DM, stage, aspirin, beta blockers, statins, neoadjuvant chemotherapy, hypertension	HR: RFS: 0.37 (0.14-0.96) OS: 0.78 (0.40-1.52)
Wang et al. 2017 (32)	Cohort	China	568/ 57.9	OC	Yes	Yes	NR	26.2±1.2	NR	DM	NR	Age, BMI, smoking, FIGO stage, pathological type and grading, postoperative residual disease, surgery type, drug delivery approaches	HR: PFS: 0.34 (0.27-0.67) OS: 0.29 (0.13-0.58)
Garcia et al. 2017 (44)	Cohort	USA	2291/ 73.2	OC	Yes	Yes	NR	NR	NR	DM	NR	Age, race, diagnosis year, stage, histology, grade, DM, total Charlson comorbidity score	OS: HR: 0.96 (0.75-1.23)
Urpilainen et al. 2018 (45)	Cohort	Finland	421/ 71	OC	Yes	Yes	26.4	NR	NR	DM	NR	Age, diagnosis year, duration of DM, stage, use of statins	CSS: HR: 1.15 (0.74-1.79)
Park et al. 2021 (46)	Cohort	South Korea	866/ NR	OC	Yes	Yes	72	NR	NR	DM	NR	Age, comorbidity level, prior use of diuretics, diagnosis year, aspirin, statins	HR: OS: 0.19 (0.07-0.53) CSS: 0.60 (0.18-2.02)
Han et al. 2015 (33)	Cohort	Canada	181/ NR	CC	Yes	Yes	60	NR	NR	DM	NR		HR: OS: 0.53 (0.27-1.07) CSS: 0.35 (0.18-0.66)
Hanprasertpong et al. 2016 (47)	Cohort	Thailand	248/ 57.8	CC	Yes	Yes	34.2	NR	NR	DM	NR	Hypertension, stage	HR: DFS: 0.53 (0.29-0.97) OS: 0.71 (0.28-1.82)
Takiuchi et al. 2017 (48)	Cohort	USA	785/ 49.2	CC	Yes	Yes	22.6	NR	NR	DM	NR	Age, stage, histology	HR: PFS: 1.11 (0.70-1.74) OS: 0.91 (0.52-1.60)

OR, odds ratio; RR, relative risk; HR, hazard ratio; CI, confidence interval; NR, not reported; OC, ovarian cancer; EC, endometrial cancer; CC, cervical cancer; BMI, body mass index; DM, diabetes mellitus; HRT, hormone replacement therapy; PCOS, polycystic ovarian syndrome; COPD, chronic obstructive pulmonary disease; WHR, waist-to-hip ratio; RFS, recurrence free survival; OS, overall survival; PFS, progression-free survival; CSS, cancer-specific survival; ASA, American society of anesthesiologists; DFS, disease-free survival.

### Results of Meta-Analysis

#### Association Between Metformin Use and Risk of Gynecologic Cancer

**Figure 2:**
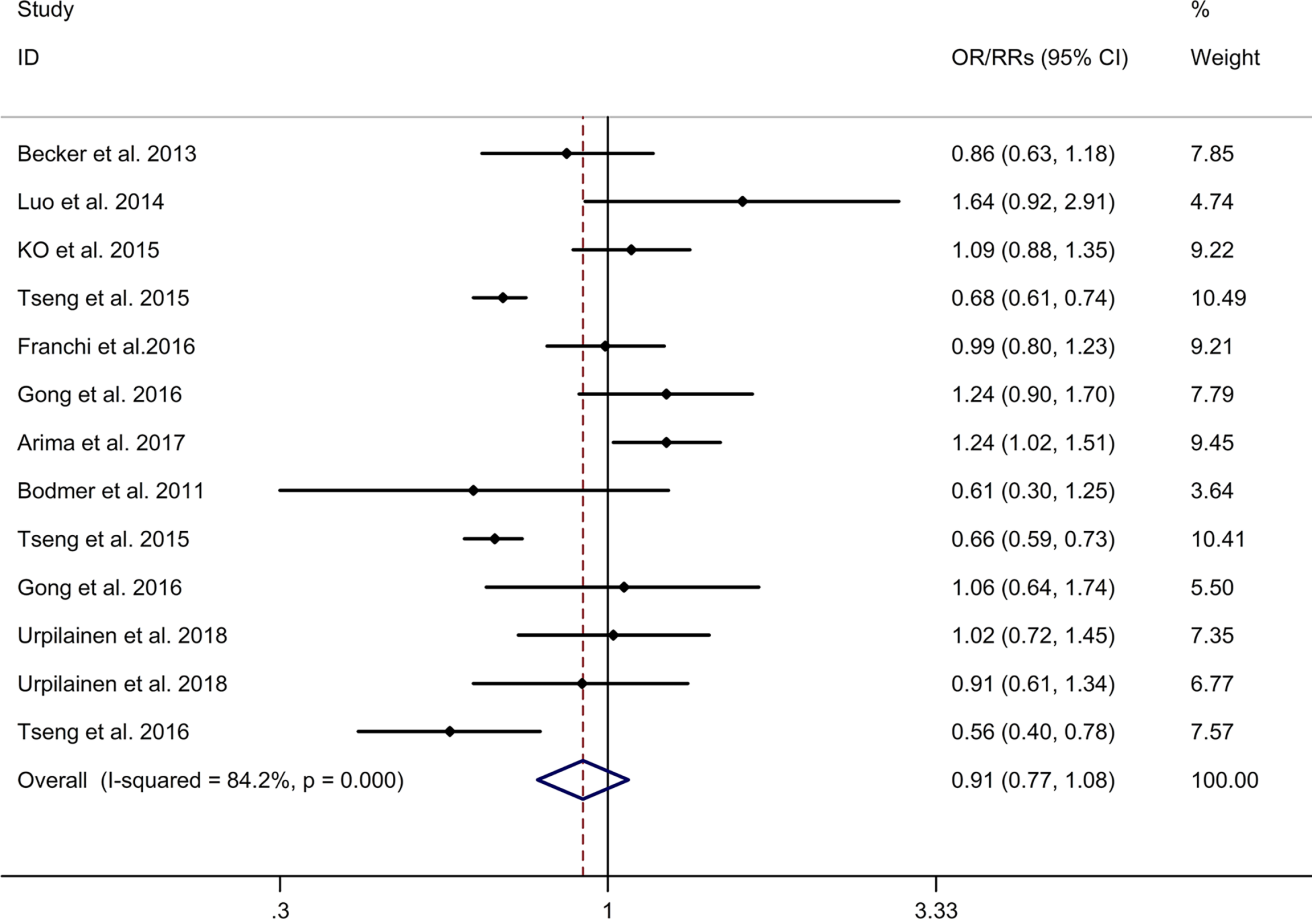
Forest plots of association between metformin use and risk of gynecologic cancer. Abbreviations: CI, confidence intervals; OR, odds ratio; RR, relative risk.

The meta-analysis showed no significant association between metformin use and risk of gynecologic cancer in DM with a random effects model (OR/RR = 0.91, 95% CI 0.77–1.08, I2 = 84.2%, p < 0.001; **Figure 2**). Meta-regression analysis showed that age of participants and publication year were not responsible for heterogeneity across studies (age of participants: p = 0.056; publication year: p = 0.967). Subgroup analysis showed no significant association between metformin use and risks of endometrial cancer, and also ovarian cancer in DM (endometrial cancer: OR/RR = 1.03, 95% CI 0.81–1.32; ovarian cancer: OR/RR = 0.82, 95% CI 0.64–1.06; **Supplementary Figure 1. A**). Subgroup analysis showed no significant association between metformin use and risks of endometrial cancer, and also ovarian cancer in DM in Caucasian (endometrial cancer: OR/RR = 1.11, 95% CI 0.97–1.26, **Supplementary Figure 2. A**; ovarian cancer: OR/RR = 0.94, 95% CI 0.76–1.18; **Supplementary Figure 2. B**). Subgroup analysis showed no significant association between metformin use and risk of endometrial cancer in DM in both cohort and case-control studies (case control: OR = 1.04, 95% CI 0.85–1.28; cohort: RR = 1.05, 95% CI 0.71–1.56; **Supplementary Figure 3. A**). Subgroup analysis showed no significant association between metformin use and risk of ovarian cancer in DM in cohort studies (RR = 0.85, 95% CI 0.59–1.22; **Supplementary Figure 3. B**). Sensitivity analysis showed no changes in the direction of effect when any one study was excluded (**Supplementary Figure 4. A**). The Begg’s test, Egger’s tests, and funnel plots showed no significant risk of publication bias (Begg’s test: p = 0.06; Egger’s test: p = 0.057; **Supplementary Figure 5. A**).

Risk of bias graph was shown in **Supplementary Figure 6. A**. Details of the risk of bias summary were shown in **Supplementary Figure 7. A**.

#### Association Between Metformin Use and OS of Gynecologic Cancer

**Figure 3:**
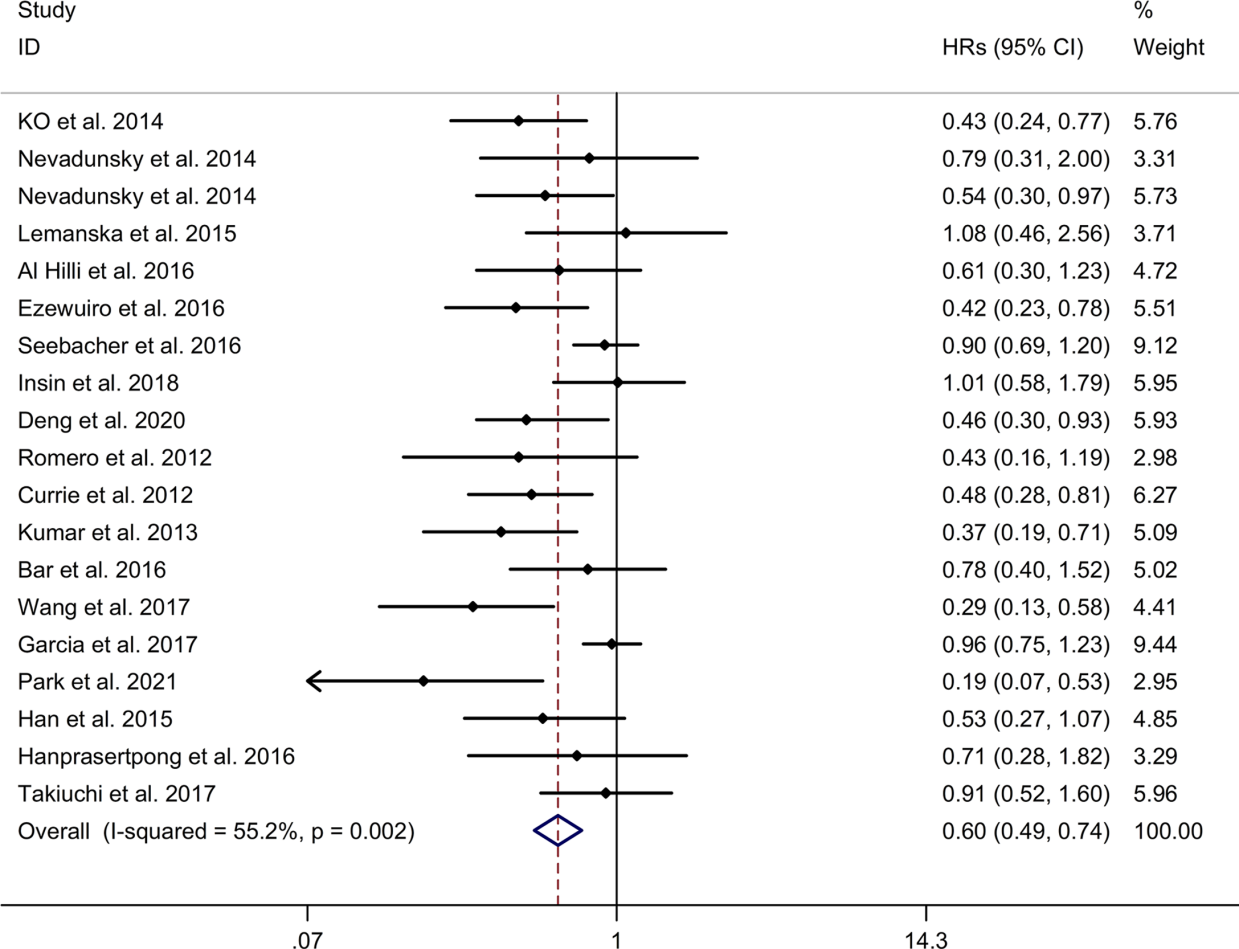
Forest plots of association between metformin use and overall survival of gynecologic cancer. Abbreviation: HR, hazard ratio.

The meta-analysis showed that metformin use was associated with a reduced OS of gynecologic cancer in DM with a random effects model (HR = 0.60, 95% CI 0.49–0.74, I2 = 55.2%, p = 0.002, **Figure 3**). Meta-regression analysis showed that age of participants and publication year were not responsible for heterogeneity across studies (age of participants: p = 0.233; publication year: p = 0.134). Subgroup analysis showed that metformin use was associated with a reduced OS of endometrial cancer and ovarian cancer in DM (endometrial cancer: HR = 0.65, 95% CI 0.50–0.85; ovarian cancer: HR = 0.47, 95% CI 0.27–0.82; **Supplementary Figure 1. B**), whereas no significant association was showed between metformin use and OS of cervical cancer in DM (HR = 0.73, 95% CI 0.49–1.08; **Supplementary Figure 1. B**). Subgroup analysis showed that metformin use was associated with a reduced OS of endometrial cancer in DM in Caucasian (HR = 0.64, 95% CI 0.48–0.87; **Supplementary Figure 2. C**). Subgroup analysis showed that metformin use was associated with a reduced OS of ovarian cancer in DM in both Caucasian and Asian populations (Caucasian: HR = 0.65, 95% CI 0.43–0.99; Asian: HR = 0.43, 95% CI 0.22–0.84; **Supplementary Figure 2. D**). Regarding the association between metformin use and OS of endometrial cancer, all included studies were designed as cohort studies. Subgroup analysis showed that metformin use was associated with a reduced OS of ovarian cancer in DM in cohort studies (HR = 0.58, 95% CI 0.40–0.86, **Supplementary Figure 3. C**). Sensitivity analysis indicated no changes in the direction of effect when any one study was excluded (**Supplementary Figure 4. B**). The Begg’s test, Egger’s tests, and funnel plots showed a significant risk of publication bias (Begg’s test: p = 0.086; Egger’s test: p = 0.003; **Supplementary Figure 5. B**).

#### Association Between Metformin Use and PFS of Gynecologic Cancer

**Figure 4:**
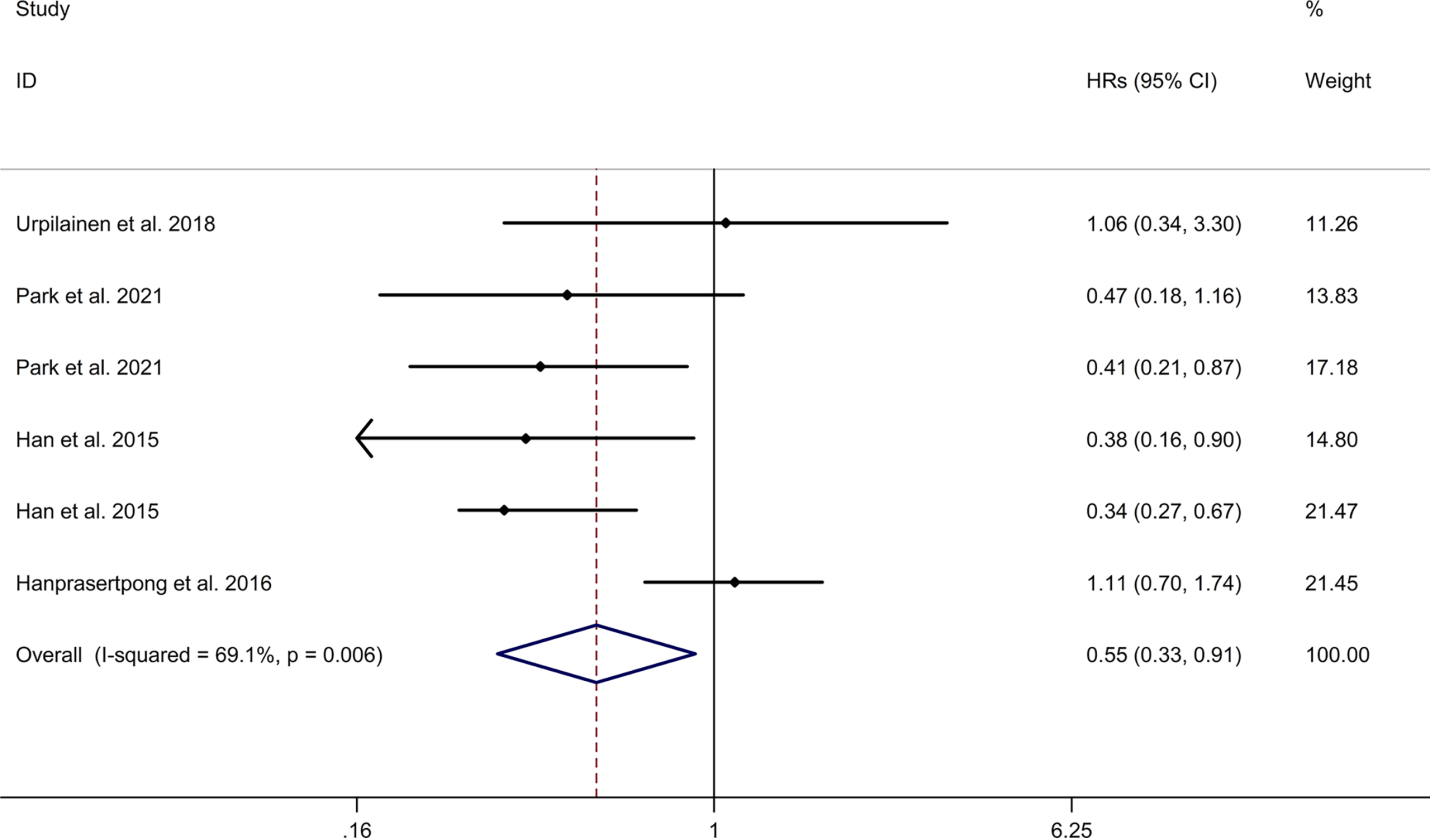
Forest plots of association between metformin use and progression-free survival of gynecologic cancer.

The meta-analysis showed that metformin use was associated with a reduced PFS of gynecologic cancer in DM with a random effects model (HR = 0.55, 95% CI 0.33–0.91, I2 = 69.1%, p = 0.006, **Figure 4**). Meta-regression analysis showed that age of participants and publication year were not responsible for heterogeneity across studies (age of participants: p = 0.490; publication year: p = 0.907). Sensitivity analysis indicated no changes in the direction of effect when any one study was excluded (**Supplementary Figure 4. C**). The Begg’s test, Egger’s tests, and funnel plots showed no significant risk of publication bias (Begg’s test: p = 0.260; Egger’s test: p = 0.881; **Supplementary Figure 5. C**).

#### Association Between Metformin Use and RFS of Gynecologic Cancer

**Figure 5:**
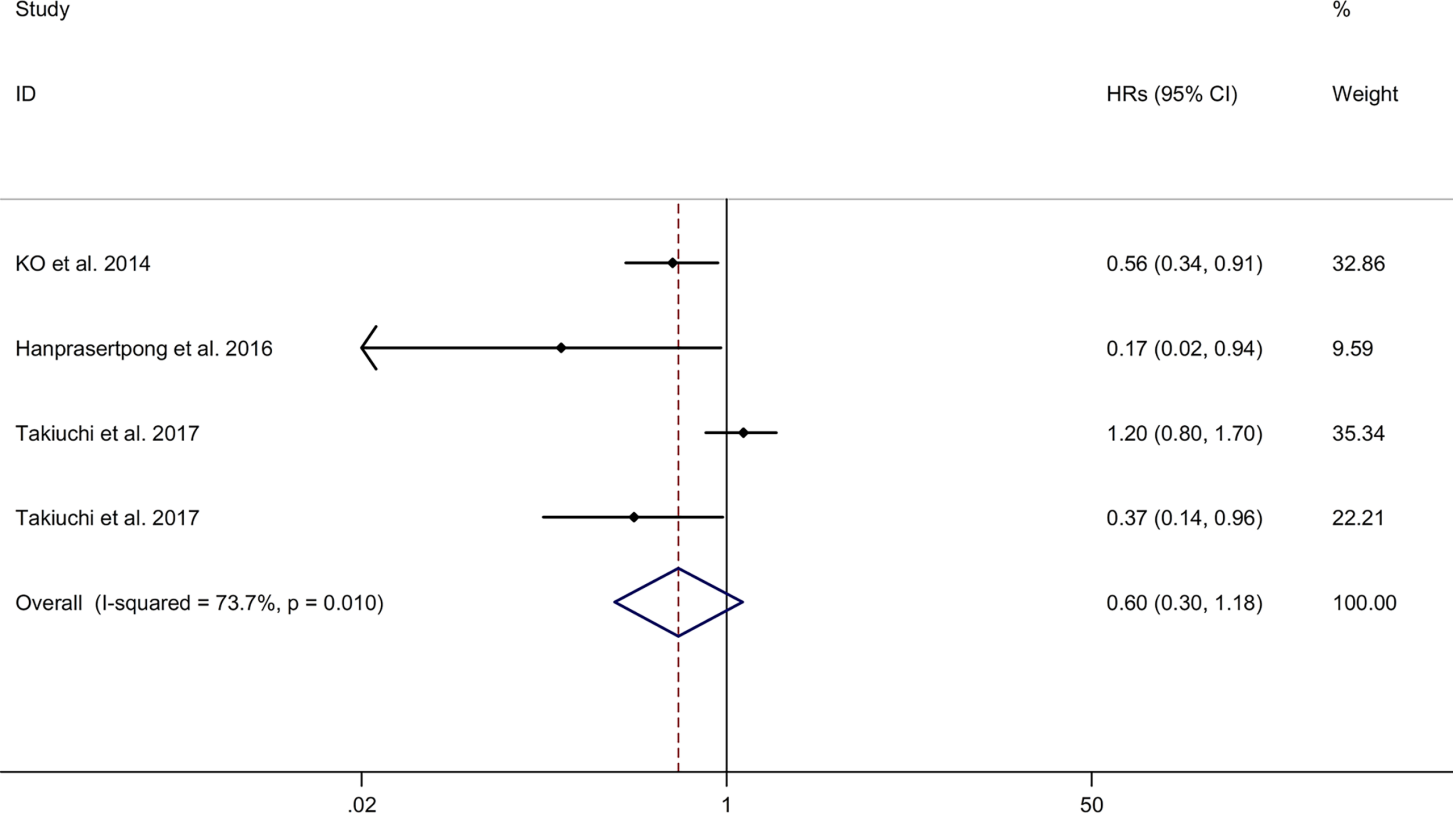
Forest plots of association between metformin use and recurrence-free survival of gynecologic cancer.

The meta-analysis showed no significant association between metformin use and RFS of gynecologic cancer in DM with a random effects model (HR = 0.60, 95% CI 0.30–1.18, I2 = 73.7%, p = 0.010, **Figure 5**). Meta-regression analysis showed that age of participants and publication year were not responsible for heterogeneity across studies (age of participants: p = 0.219; publication year: p = 0.765). Subgroup analysis showed no significant association between metformin use and RFS of endometrial cancer in DM (HR = 0.68, 95% CI 0.31–1.49; **Supplementary Figure 1. C**). Sensitivity analysis indicated no changes in the direction of effect when any one study was excluded (**Supplementary Figure 4. D**). The Begg’s test, Egger’s tests, and funnel plots showed no significant risk of publication bias (Begg’s test: p = 1.000; Egger’s test: p = 0.186; **Supplementary Figure 5. D**).

#### Association Between Metformin Use and CSS of Gynecologic Cancer

**Figure 6:**
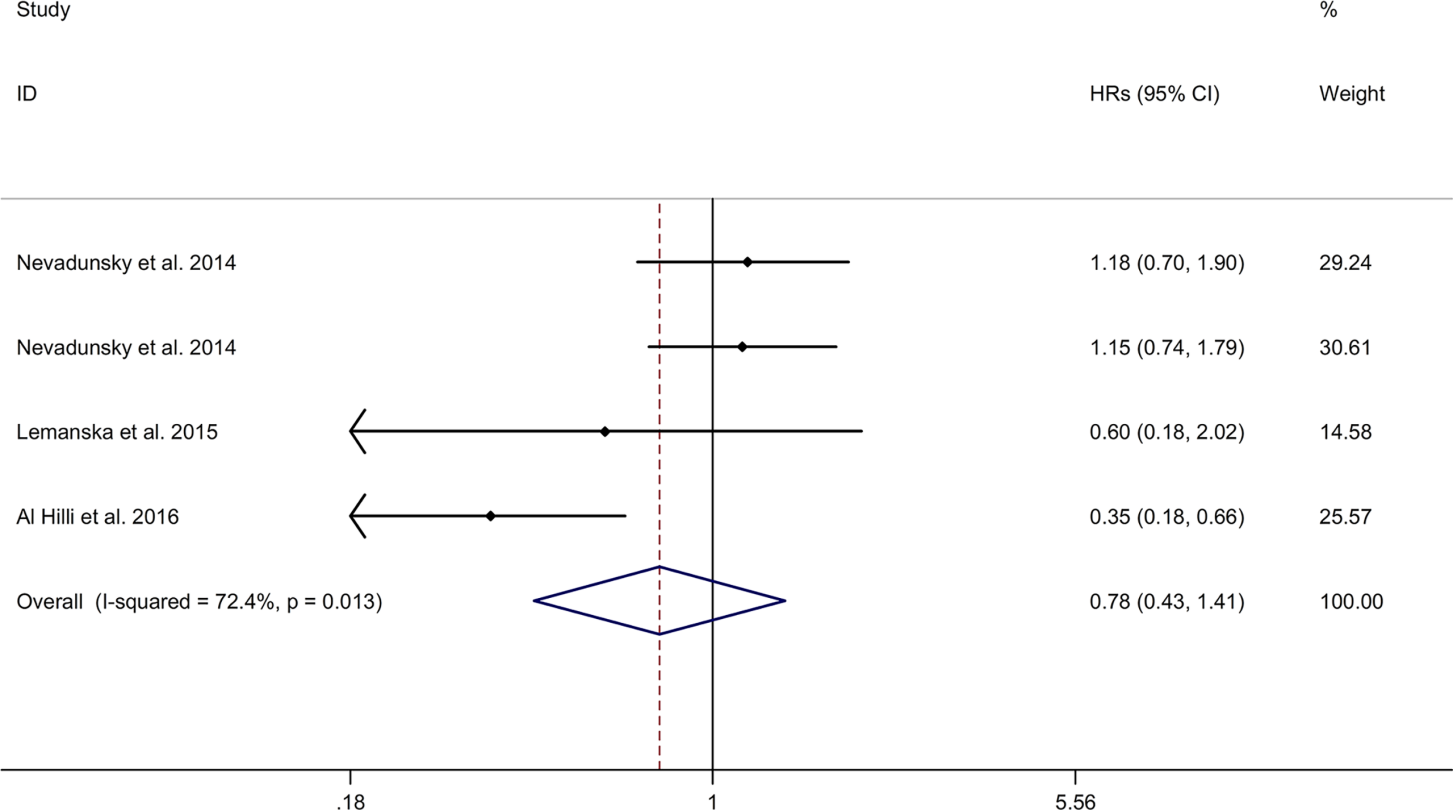
Forest plots of association between metformin use and cancer-specific survival of gynecologic cancer.

The meta-analysis showed no significant association between metformin use and CSS of gynecologic cancer in DM with a random effects model (HR = 0.78, 95% CI 0.43–1.41, I2 = 72.4%, p = 0.013, **Figure 6**). Meta-regression analysis showed that publication year was not responsible for heterogeneity across studies (p = 0.776). Sensitivity analysis indicated no changes in the direction of effect when any one study was excluded (**Supplementary Figure 4. E**). The Begg’s test, Egger’s tests, and funnel plots showed no significant risk of publication bias (Begg’s test: p = 0.308; Egger’s test: p = 0.431; **Supplementary Figure 5. E**).

Risk of bias graph was shown in **Supplementary Figure 6. B**. Details of the risk of bias summary were shown in **Supplementary Figure 7. B**.

## Discussion

### Association Between Metformin Use and Risk of Gynecologic Cancer

In this systemic review and meta-analysis, we all included 31 published articles (11 articles for the risk and 20 articles for the prognosis of gynecological cancer). In this meta-analysis, we found that there were no significant associations between metformin use and reduced risk of gynecological cancer in DM (OR/RR = 0.91, 95% CI: 0.77–1.08). No associations were also showed between metformin and risk of endometrial cancer or ovarian cancer in DM in subgroup analysis (endometrial cancer: OR/RR = 1.03, 95% CI: 0.81–1.32; ovarian cancer: OR/RR = 0.82, 95% CI: 0.64–1.06). These results were different from results of previous meta-analysis. Wen et al. reported that metformin use was associated with a lower risk of gynecological cancer based on seven included studies, with a 51% decrease (RR = 0.49, 95% CI: 0.29–0.82) (34). Shi et al. also showed a significant reduction in risk of ovarian cancer among metformin users (OR = 0.76, 95% CI: 0.62–0.93) (33). We also noticed that some results had similar views. The pooled results of seven studies suggested that there was no association between metformin and endometrial cancer risk (OR = 1.05, 95% CI: 0.82–1.35) (32). A meta-analysis discussing the relationship between metformin use and risk of cancer among T2DM patients indicated that neither endometrial cancer risk nor ovarian cancer risk was associated with metformin use (endometrial cancer: OR = 1.11, 95% CI: 0.65–1.88; ovarian cancer: OR = 0.78, 95% CI: 0.53–1.15) (59). The present meta-analysis is an updated study for previous meta-analyses. In addition, the present study systematically explored association between metformin use and risk of different types of gynecologic cancer.

High heterogeneity was showed between studies exploring the association between metformin use and risk of gynecologic cancer. However, subgroup or meta-regression analysis did not identify the sources of heterogeneity across studies. The study included observational studies, which were inhomogeneous both clinically and methodologically. Thus, high heterogeneity is not surprising. Heterogeneities in clinical features, such as age, ethnicities, diabetes duration, follow-up duration, different dosage of metformin, and adjusted variables, might be the sources of heterogeneity across studies. However, most of the studies included in the meta-analysis did not provide sufficient information for these features, such as diabetes duration and different dosage of metformin. Meta-regression analysis could not be conducted due to the insufficient information for these features. In addition, an amount of the included studies were retrospectively designed, which might cause recall and selection bias.

### Association Between Metformin Use and Prognosis of Gynecologic Cancer

Additionally, we appraised the effect of metformin on the prognosis of gynecological cancer. The pooled data provided that significantly improved OS of gynecological cancer was observed in metformin users compared to non-user, similar as endometrial cancer and ovarian cancer in subgroup analysis (gynecological cancer: HR = 0.60, 95% CI: 0.49–0.74; endometrial cancer: HR = 0.65, 95% CI: 0.50–0.85; ovarian cancer: HR = 0.47, 95% CI: 0.27–0.82). Metformin therapy was associated with a 45% reduction in PFS of gynecological cancer (HR = 0.55, 95% CI: 0.33–0.91). Chu et al. also reported that metformin was associated with a better OS and a lower risk of recurrence in endometrial cancer patients (OS: HR = 0.61, 95% CI: 0.48–0.77; recurrence: HR: 0.50, 95% CI: 0.28–0.92) (32). The pooled data of seven studies showed that a significant reduction of mortality and a prolonged PFS associated with the use of metformin were found among ovarian cancer patients (OR = 0.55, 95% CI: 0.36–0.84) (33). These results were consistent with our findings. The present meta-analysis is an updated study for previous meta-analyses. In addition, the present study systematically explored association between metformin use and prognosis of different types of gynecologic cancer.

High heterogeneity is one of the potential problems when clarifying the results of the meta-analysis. Although the present study has used the explicit criteria for study inclusion and exclusion, performing data extraction, and statistical analysis strictly, the high heterogeneity between studies still existed. The high heterogeneity might be caused by features of participants and clinical characteristics.

It should be noted that the meta-analysis mainly computed the results of observational studies, which were unavoidably prone to bias and confounding inherent in the study design. Thus, the potential effects of metformin on gynecologic cancer need to be identified by randomized controlled trials (RCTs). RCTs were essential to explore the beneficial effects of metformin on gynecologic cancer.

### Mechanism Studies

The anticancer effect of metformin has been proved in vitro cell system. Zou et al. reported that metformin can suppress proliferation and induce apoptosis SKOV3 ovarian cancer cells involving metastasis-associated 1 (60). Cui et al. showed that the combined use of metformin and RG7388 can significantly inhibit cell growth and increase apoptosis of A2780 and SKOV3 cells via the phosphoinositide 3-kinase (PI3K)/AKT/mTOR pathway while enhancing the accumulation of intracellular ROS (61). Besides, metformin can reduce mesothelin expression, subsequently induce the expression of VEGF and TGFβ1, and finally impair the capillary-like structure formation capacity of SKOV3 cells (62). Metformin can inhibit the proliferation of endometrial cancer cell lines Ishikawa and RL95-2 by suppressing programmed death-ligand 1 and activating AMPK signaling (63). Qiang et al. reported that metformin can inhibit the activation of PI3K/AKT/murine double minute 2 signaling, resulting in the suppression of Ishikawa cells proliferation and migration (64). In addition, metformin has been shown to have anticarcinogenic activity for gynecological cancers in vivo. Lengyel et al. (20) reported that metformin prevents tumor growth and increases sensitivity to chemotherapy in mouse models. Rattan et al. (21) found that, except for inhibiting tumor cell proliferation, metformin use inhibits both angiogenesis and metastatic spread of ovarian cancer in vivo.

### Limitations

Nevertheless, there were some limitations in this meta-analysis that need to be addressed. First, we tried our best to collect the published articles in English, the data from articles published in other languages or unpublished may be missed. Second, we noticed that, in all 31 included articles, there were only 3 articles involving the association between cervical cancer and metformin use. Last, we need more detail information to accurately evaluate the association including drug dose, duration, and risk factors such as smoking and alcohol intake.

## Conclusion

In conclusion, this meta-analysis indicated that metformin may be a useful adjuvant agent for gynecological cancer with DM, especially for patients with ovarian cancer and endometrial cancer. However, the safety and efficacy of metformin among non-diabetic cancer and cervical cancer patients should be treated with caution. More well-designed, large-sample studies are needed to rigorously evaluate in the future.

## Data Availability Statement

The original contributions presented in the study are included in the article/**Supplementary Material**. Further inquiries can be directed to the corresponding author.

## Author Contributions

KY: Study design, manuscript writing, data collection, data analysis, software use. HZ: Data collection, data analysis. TL: Study design, manuscript writing and revision, data collection, data analysis, software use, supervision. All authors read and approved the final version of the manuscript.

## Conflict of Interest

The authors declare that the research was conducted in the absence of any commercial or financial relationships that could be construed as a potential conflict of interest.

## Publisher’s Note

All claims expressed in this article are solely those of the authors and do not necessarily represent those of their affiliated organizations, or those of the publisher, the editors and the reviewers. Any product that may be evaluated in this article, or claim that may be made by its manufacturer, is not guaranteed or endorsed by the publisher.
