# Supplementary material for: Association Between Metformin Use and the Risk, Prognosis of Gynecologic Cancer
Source: Front Oncol. 2022 Jul 11;12:942380. doi: 10.3389/fonc.2022.942380 (PMC9309370; doi:10.3389/fonc.2022.942380)
Supplement: Supplementary file 1 [file DataSheet_1.docx]

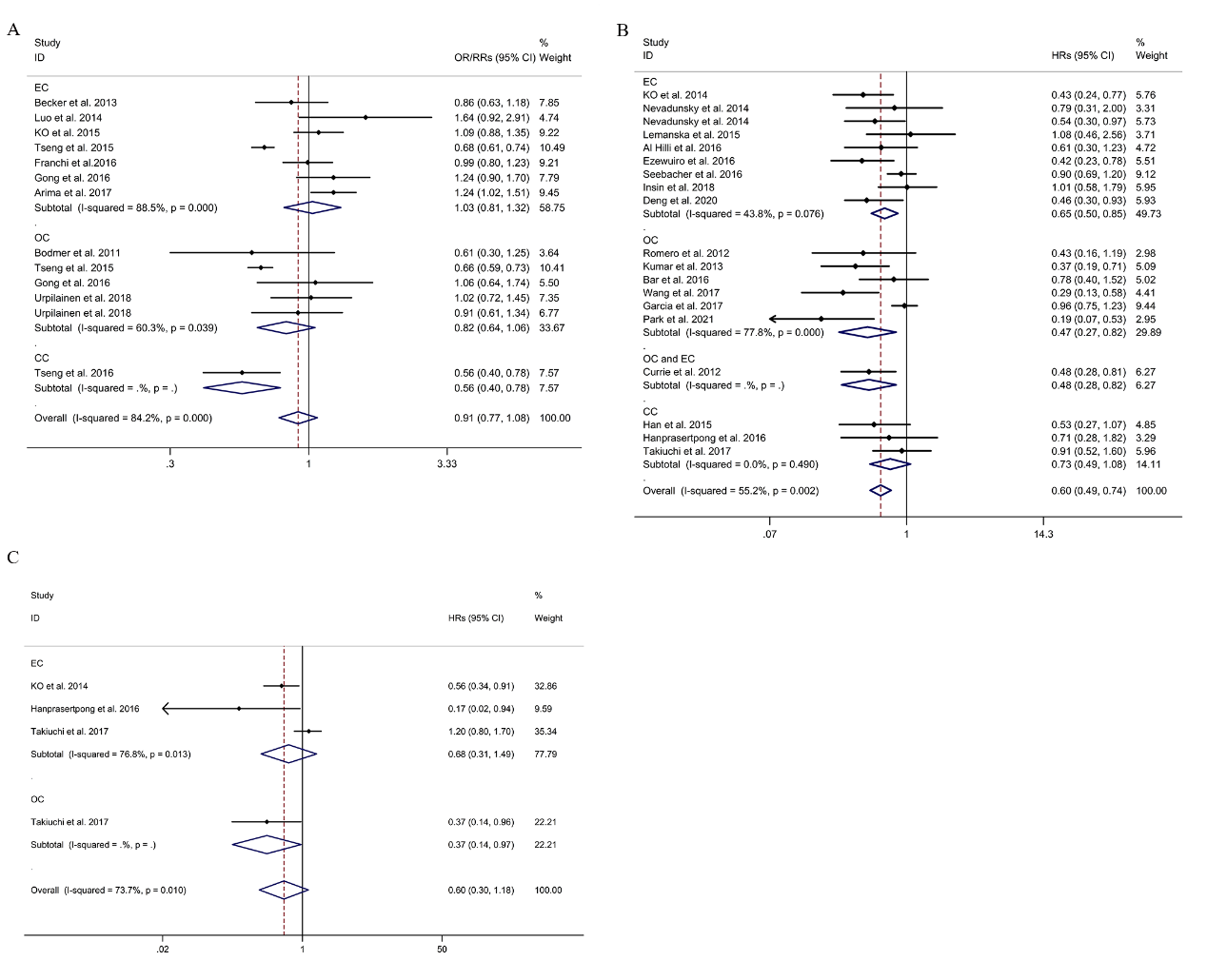


Supplementary figure 1. Subgroup studies regarding association between metformin use and risk of different types of gynecologic cancer (A), overall survival of different types of gynecologic cancer (B), recurrence free survival of different types of gynecologic cancer (C). Abbreviations: CC, cervical cancer; CI, confidence intervals; EC, endometrial cancer; HR, hazard ratio; OC, ovarian cancer; OR, odds ratio; RR, relative risk.
