# Supplementary material for: Association Between Metformin Use and the Risk, Prognosis of Gynecologic Cancer
Source: Front Oncol. 2022 Jul 11;12:942380. doi: 10.3389/fonc.2022.942380 (PMC9309370; doi:10.3389/fonc.2022.942380)
Supplement: Supplementary file 2 [file DataSheet_2.docx]

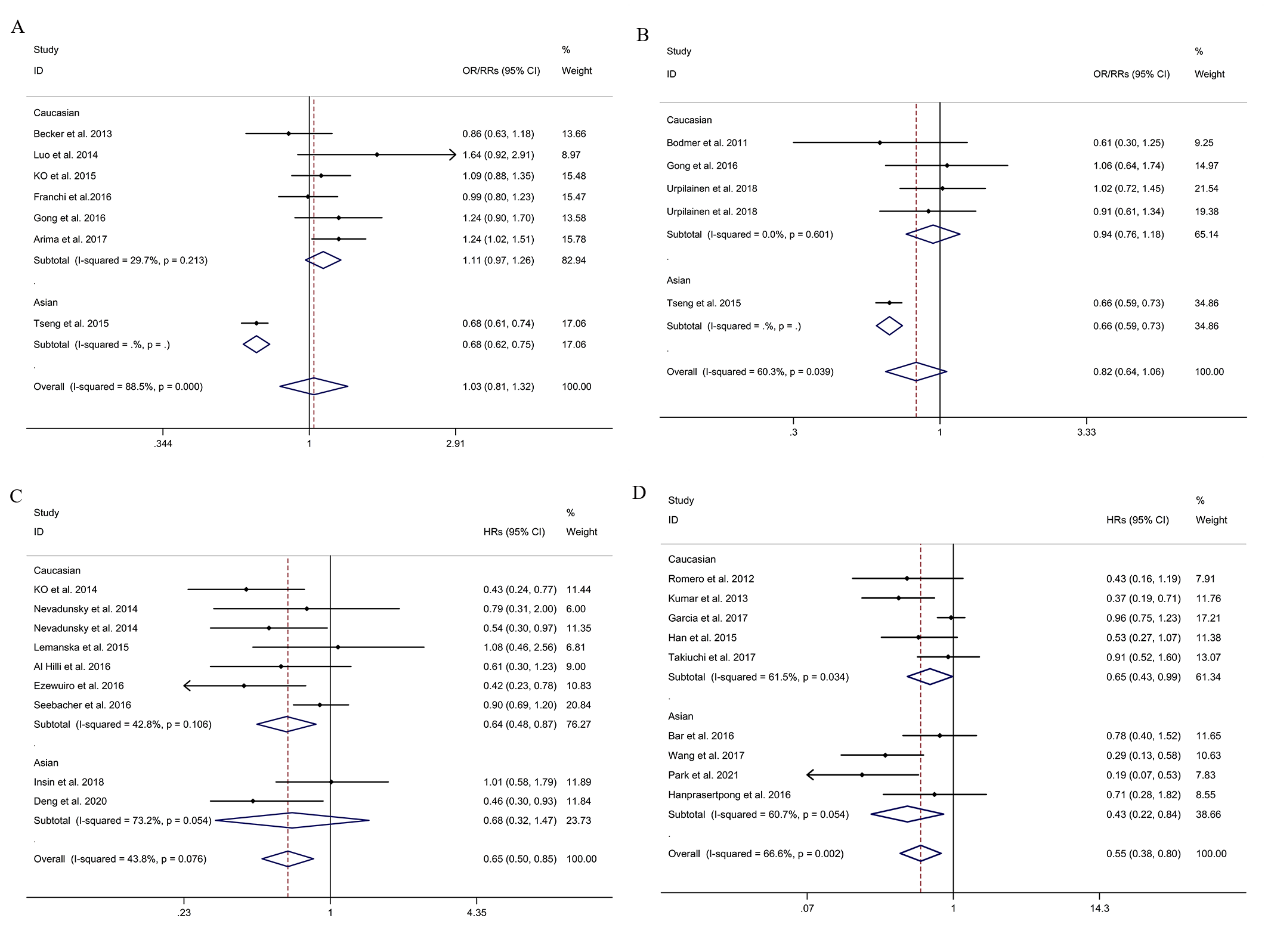


Supplementary figure 2. Subgroup studies regarding association between metformin use and risk of endometrial cancer in different ethnicities (A), risk of ovarian cancer in different ethnicities (B), overall survival of endometrial cancer in different ethnicities (C), overall survival of ovarian cancer in different ethnicities (D). Abbreviations: CI, confidence intervals; HR, hazard ratio; OR, odds ratio; RR, relative risk.
