# Supplementary material for: Association Between Metformin Use and the Risk, Prognosis of Gynecologic Cancer
Source: Front Oncol. 2022 Jul 11;12:942380. doi: 10.3389/fonc.2022.942380 (PMC9309370; doi:10.3389/fonc.2022.942380)
Supplement: Supplementary file 3 [file DataSheet_3.docx]

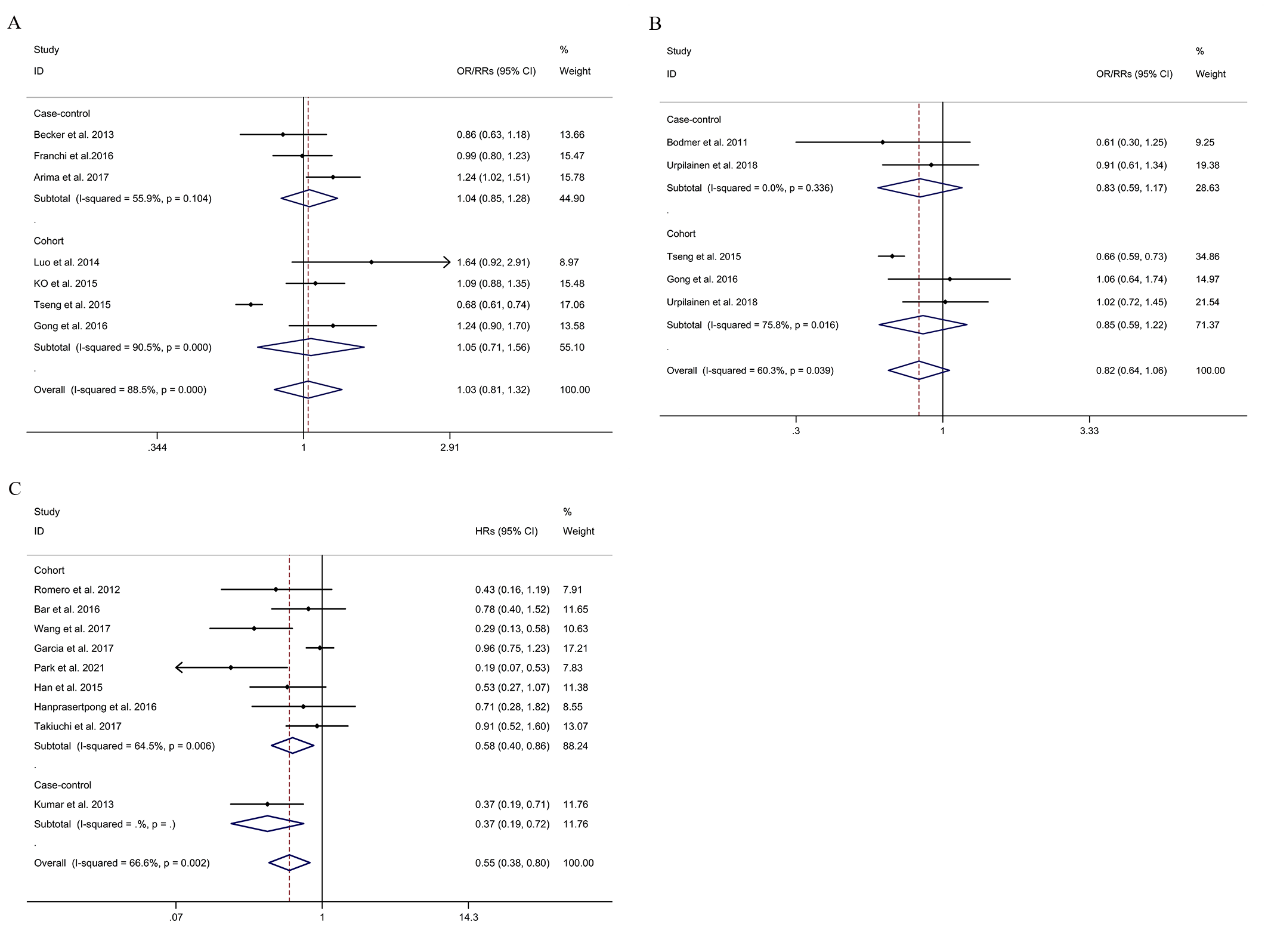


Supplementary figure 3. Subgroup studies regarding association between metformin use and risk of endometrial cancer in different study types (A), risk of ovarian cancer in different study types (B), overall survival of ovarian cancer in different study types (C). Abbreviations: CI, confidence intervals; HR, hazard ratio; OR, odds ratio; RR, relative risk.
