# Supplementary material for: Association Between Metformin Use and the Risk, Prognosis of Gynecologic Cancer
Source: Front Oncol. 2022 Jul 11;12:942380. doi: 10.3389/fonc.2022.942380 (PMC9309370; doi:10.3389/fonc.2022.942380)
Supplement: Supplementary file 4 [file DataSheet_4.docx]

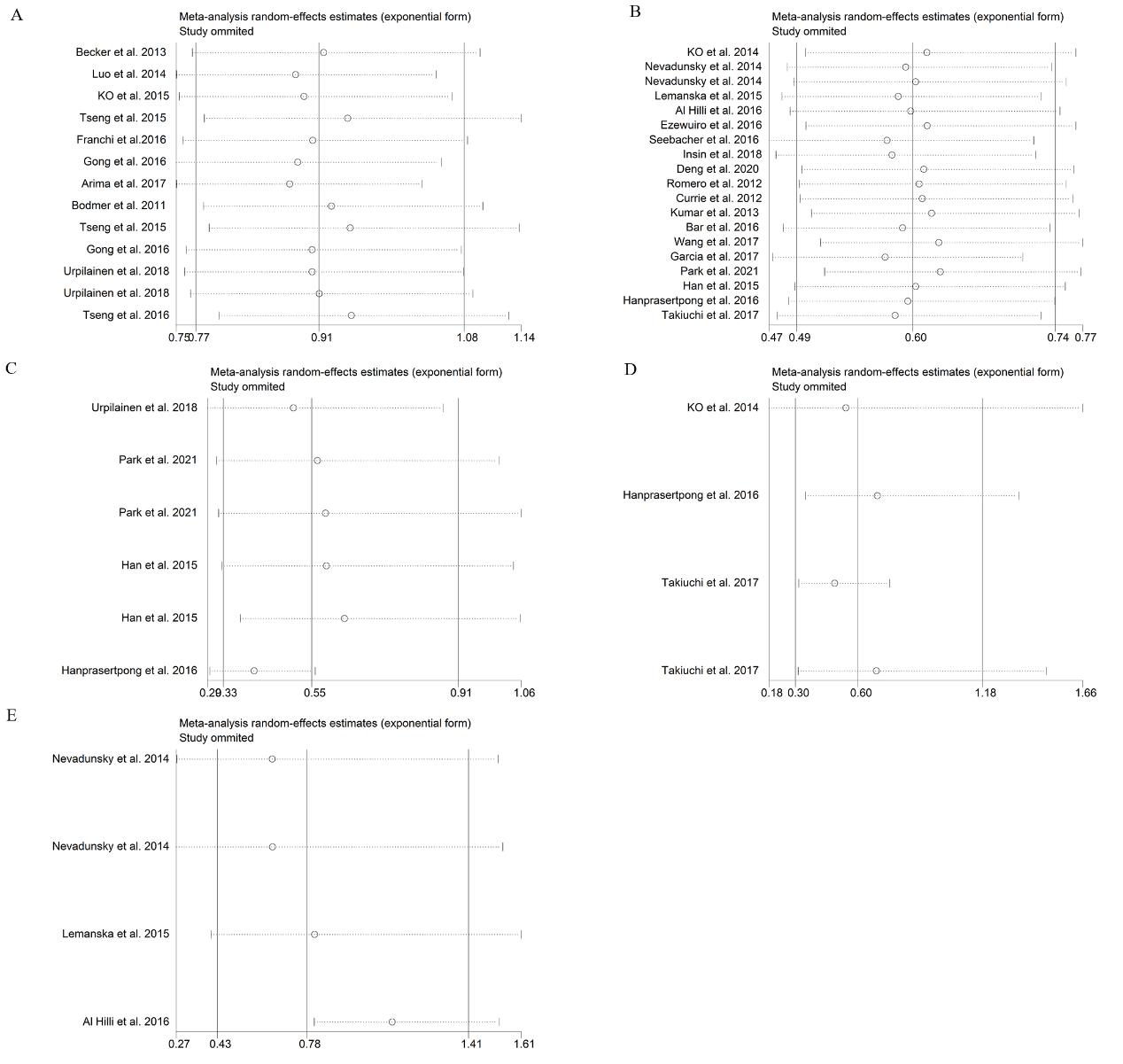


Supplementary figure 4. Sensitivity analysis regarding association between metformin use and risk of gynecologic cancer (A), overall survival of gynecologic cancer (B), progression-free survival of gynecologic cancer (C), recurrence free survival of gynecologic cancer (D), cancer-specific survival of gynecologic cancer (E).
