# Supplementary figures and images for: Association Between Metformin Use and the Risk, Prognosis of Gynecologic Cancer
Source: Front Oncol. 2022 Jul 11;12:942380. doi: 10.3389/fonc.2022.942380 (PMC9309370; doi:10.3389/fonc.2022.942380)

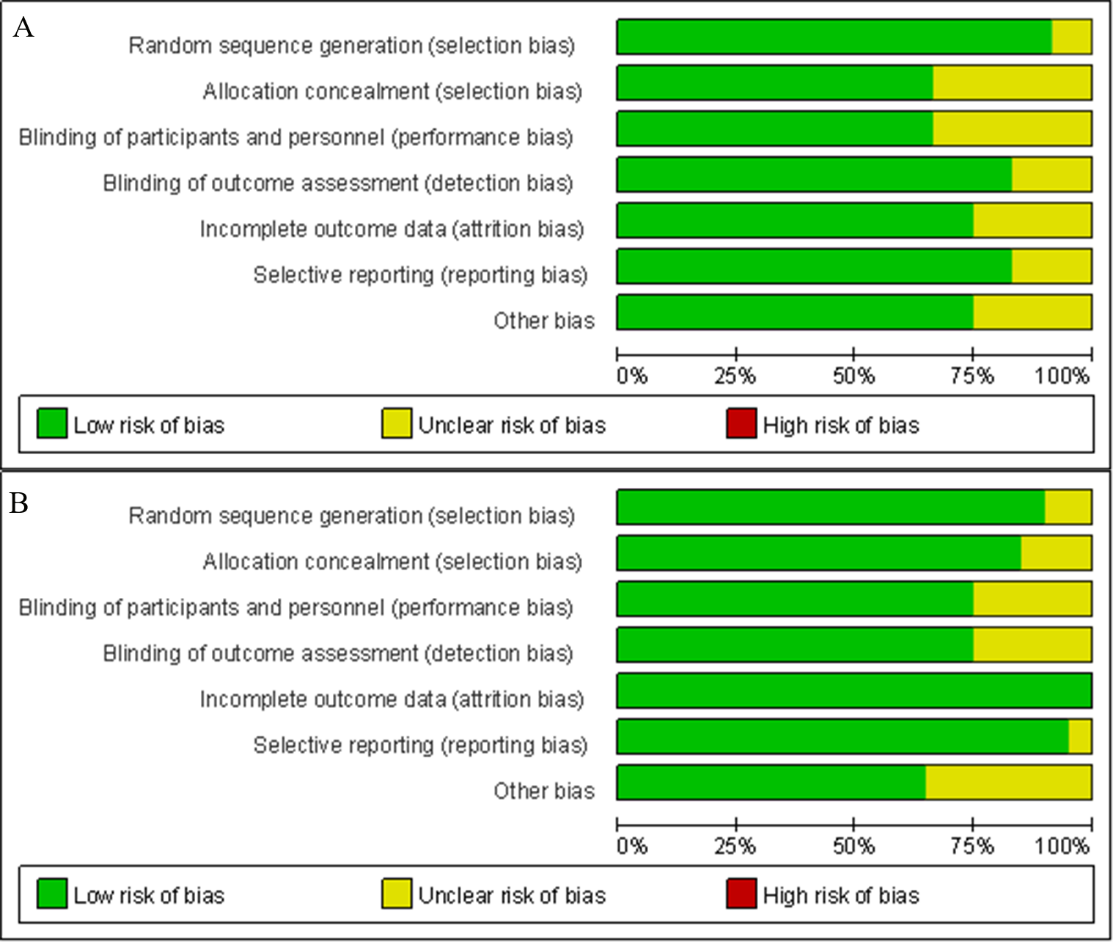


Supplementary figure 6. Risk of bias graph

Supplement: Supplementary file 6 [file DataSheet_6.docx]

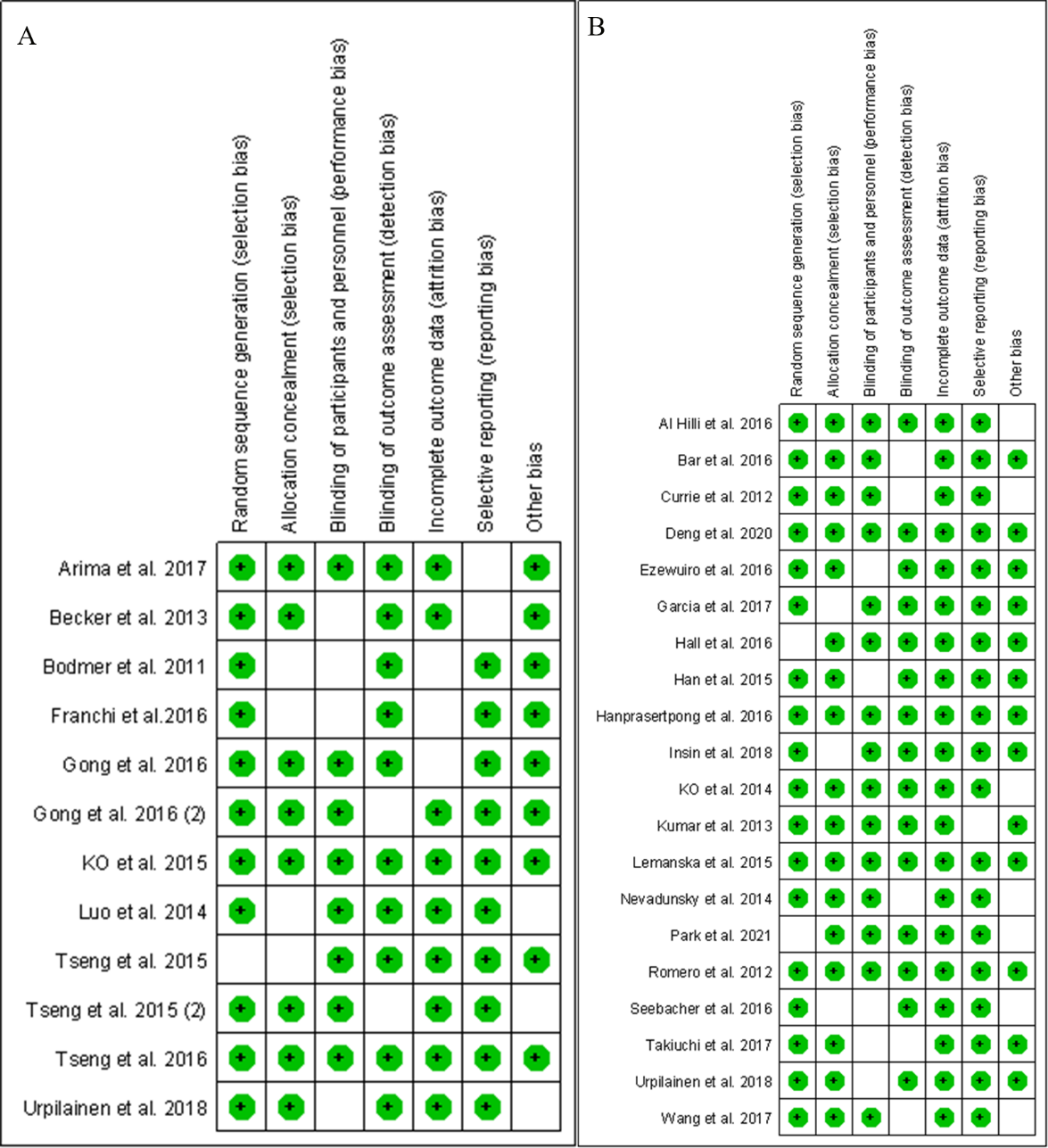


Supplementary figure 7. Risk of bias summary.

Supplement: Supplementary file 7 [file DataSheet_7.docx]
