# Supplementary material for: Association Between Metformin Use and the Risk, Prognosis of Gynecologic Cancer
Source: Front Oncol. 2022 Jul 11;12:942380. doi: 10.3389/fonc.2022.942380 (PMC9309370; doi:10.3389/fonc.2022.942380)
Supplement: Supplementary file 8 [file Table_1.docx]

Supplementary table 1. Search Query.

| Step | Search strategy |
| --- | --- |
| #1 | Search (("gynaecologic"[All Fields] OR "gynecologic"[All Fields] OR "gynecologically"[All Fields] OR "gynecology"[MeSH Terms] OR "gynecology"[All Fields] OR "gynaecological"[All Fields] OR "gynecological"[All Fields]) AND ("cancer s"[All Fields] OR "cancerated"[All Fields] OR "canceration"[All Fields] OR "cancerization"[All Fields] OR "cancerized"[All Fields] OR "cancerous"[All Fields] OR "neoplasms"[MeSH Terms] OR "neoplasms"[All Fields] OR "cancer"[All Fields] OR "cancers"[All Fields])) OR ("ovarian neoplasms"[MeSH Terms] OR ("ovarian"[All Fields] AND "neoplasms"[All Fields]) OR "ovarian neoplasms"[All Fields] OR ("ovarian"[All Fields] AND "cancer"[All Fields]) OR "ovarian cancer"[All Fields]) OR "oophoroma"[All Fields] OR ("ovarian neoplasms"[MeSH Terms] OR ("ovarian"[All Fields] AND "neoplasms"[All Fields]) OR "ovarian neoplasms"[All Fields] OR ("ovary"[All Fields] AND "carcinoma"[All Fields]) OR "ovary carcinoma"[All Fields]) OR ("carcinoma, ovarian epithelial"[MeSH Terms] OR ("carcinoma"[All Fields] AND "ovarian"[All Fields] AND "epithelial"[All Fields]) OR "ovarian epithelial carcinoma"[All Fields] OR ("carcinoma"[All Fields] AND "ovary"[All Fields]) OR "carcinoma of the ovary"[All Fields]) OR ("endometrial neoplasms"[MeSH Terms] OR ("endometrial"[All Fields] AND "neoplasms"[All Fields]) OR "endometrial neoplasms"[All Fields] OR ("endometrial"[All Fields] AND "cancer"[All Fields]) OR "endometrial cancer"[All Fields]) OR ("endometrial neoplasms"[MeSH Terms] OR ("endometrial"[All Fields] AND "neoplasms"[All Fields]) OR "endometrial neoplasms"[All Fields] OR ("endometrial"[All Fields] AND "carcinoma"[All Fields]) OR "endometrial carcinoma"[All Fields]) OR ("endometrial neoplasms"[MeSH Terms] OR ("endometrial"[All Fields] AND "neoplasms"[All Fields]) OR "endometrial neoplasms"[All Fields] OR ("carcinoma"[All Fields] AND "endometrium"[All Fields]) OR "carcinoma of the endometrium"[All Fields]) OR (("endometrial neoplasms"[MeSH Terms] OR ("endometrial"[All Fields] AND "neoplasms"[All Fields]) OR "endometrial neoplasms"[All Fields] OR ("endometrial"[All Fields] AND "carcinoma"[All Fields]) OR "endometrial carcinoma"[All Fields]) AND ("uterus"[MeSH Terms] OR "uterus"[All Fields] OR "uteri"[All Fields])) OR ("uterine cervical neoplasms"[MeSH Terms] OR ("uterine"[All Fields] AND "cervical"[All Fields] AND "neoplasms"[All Fields]) OR "uterine cervical neoplasms"[All Fields] OR ("cervical"[All Fields] AND "cancer"[All Fields]) OR "cervical cancer"[All Fields]) OR (("cervic"[All Fields] OR "cervicals"[All Fields] OR "cervices"[All Fields] OR "neck"[MeSH Terms] OR "neck"[All Fields] OR "cervical"[All Fields] OR "uterine cervicitis"[MeSH Terms] OR ("uterine"[All Fields] AND "cervicitis"[All Fields]) OR "uterine cervicitis"[All Fields] OR "cervicitis"[All Fields]) AND ("carcinoma"[MeSH Terms] OR "carcinoma"[All Fields] OR "carcinomas"[All Fields] OR "carcinoma s"[All Fields])) OR (("carcinoma"[MeSH Terms] OR "carcinoma"[All Fields] OR "carcinomas"[All Fields] OR "carcinoma s"[All Fields]) AND ("cervix uteri"[MeSH Terms] OR ("cervix"[All Fields] AND "uteri"[All Fields]) OR "cervix uteri"[All Fields] OR ("uterine"[All Fields] AND "cervix"[All Fields]) OR "uterine cervix"[All Fields])) |
| #2 | Search "metformin"[MeSH Terms] OR "metformin"[All Fields] OR "metformine"[All Fields] OR "metformin s"[All Fields] OR "metformins"[All Fields] |
| #3 | (#1) AND (#2) |
